# Improving Lesion Location Reproducibility in Handheld Breast Ultrasound

**DOI:** 10.3390/diagnostics14151602

**Published:** 2024-07-25

**Authors:** James Chiu, Davide Bova, Georgia Spear, Jacob Ecanow, Alyssa Choate, Pierre Besson, Calin Caluser

**Affiliations:** 1 Department of Radiology, Endeavor Health, 2650 Ridge Ave, Evanston, IL 60201, USA; 2 Dacia Medical Clinic, 917 S Oak Park Ave, Suite B, Oak Park, IL 60304, USA; 3 Department of Radiology, Loyola University Medical Center, 2160 S First Ave, Maywood, IL 60153, USA; 4 MetriTrack Inc., 4415 Harrison St., #243, Hillside, IL 60162, USA; 5 Midwest Center for Advanced Imaging, Rush University Medical System, 4355 Montgomery Rd, Naperville, IL 60564, USA

**Keywords:** breast ultrasound, navigation tool, breast lesion mapping, reproducibility

## Abstract

Interoperator variability in the reproducibility of breast lesions found by handheld ultrasound (HHUS) can significantly interfere with clinical care. This study analyzed the features associated with breast mass position differences during HHUS. The ability of operators to reproduce the position of small masses and the time required to generate annotations with and without a computer-assisted scanning device (DEVICE) were also evaluated. This prospective study included 28 patients with 34 benign or probably benign small breast masses. Two operators generated manual and automated position annotations for each mass. The probe and body positions were systematically varied during scanning with the DEVICE, and the features describing mass movement were used in three logistic regression models trained to discriminate small from large breast mass displacements (cutoff: 10 mm). All models successfully discriminated small from large breast mass displacements (areas under the curve: 0.78 to 0.82). The interoperator localization precision was 6.6 ± 2.8 mm with DEVICE guidance and 19.9 ± 16.1 mm with manual annotations. Computer-assisted scanning reduced the time to annotate and reidentify a mass by 33 and 46 s on average, respectively. The results demonstrated that breast mass location reproducibility and exam efficiency improved by controlling operator actionable features with computer-assisted HHUS.

## 1. Introduction

Breast ultrasound is a powerful diagnostic tool and useful in guiding interventional breast procedures. It has been increasingly used to screen women with dense breasts and can increase breast cancer detection rates by 1.9–4.2/1000 when added to 2D digital mammography or tomosynthesis [1]. About half of women over 40 years old have dense breasts [2] and the risk for developing cancer is increased by up to 4–6 times in women with extremely dense breasts [3]. The early detection of subcentimeter cancers is essential for the successful and cost-effective treatment of breast cancer [4]. The five-year cancer survival for stage 1 patients is over 98% and drops to 22% at stage 4 [5].

Despite continuous advances in image acquisition and post-processing, handheld ultrasound remains limited by operator dependence, variable reproducibility, length of examination, and the shortage of dedicated technologists and breast radiologists [6,7,8]. Whole-breast ultrasound screening relies on the assumption that incidental probably benign lesions can be successfully followed [8]. The consistent reporting of the lesion location, size, description and depiction of features is required to reproduce lesions at follow-up ultrasound exams [8]. Nevertheless, these requirements are not followed by all ultrasound operators; a published study showed that 60.5% of cases from 86 institutions did not fully meet all American College of Radiology (ACR) standards for breast ultrasound exams [9]. 

The ability to identify subcentimeter lesions in a reproducible and consistent manner has a major impact in disease management. It is especially important since the goal of breast cancer screening is to find cancers 10 mm or smaller, which is when they have a good prognosis [8]. The lack of reproducibility in lesion characterization in breast ultrasound is particularly important for small lesions [6]. Despite high reliability across readers for the clock face position (CFP) annotations, distance to nipple (DN) annotations and lesion size measurements, the overall reproducibility of subcentimeter lesions and the inter-observer agreement were significantly lower than for larger lesions [6,8]. Most breast lesions are reported to be less than a centimeter (89% [8] and 65% [6]). The CFP, DN and probe orientation specification currently used to annotate the position of breast ultrasound images use a 2D polar coordinate system. These annotations do not provide depth or probe compression information and the described search area may cover a few centimeters, especially when away from the nipple, which may interfere with the identification of small lesions. 

**Figure A1:**
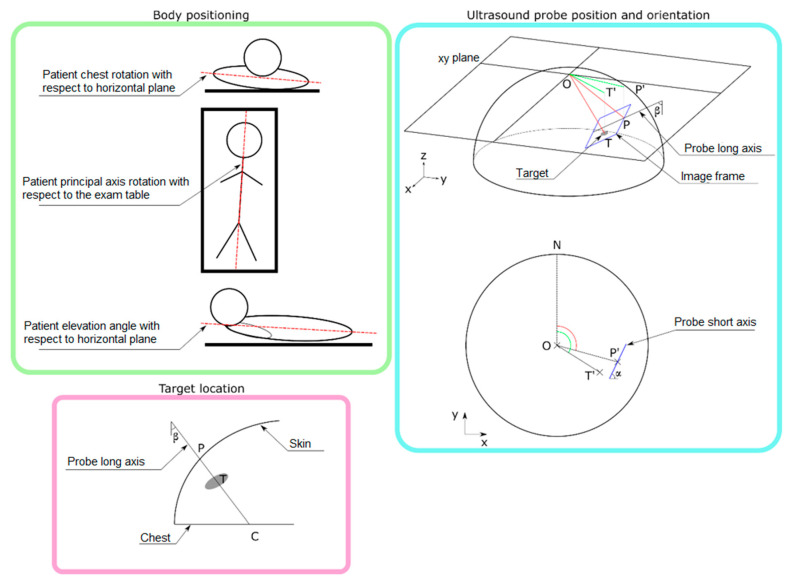
Representation of the landmarks used by the DEVICE during a breast ultrasound exam.

**Figure A2:**
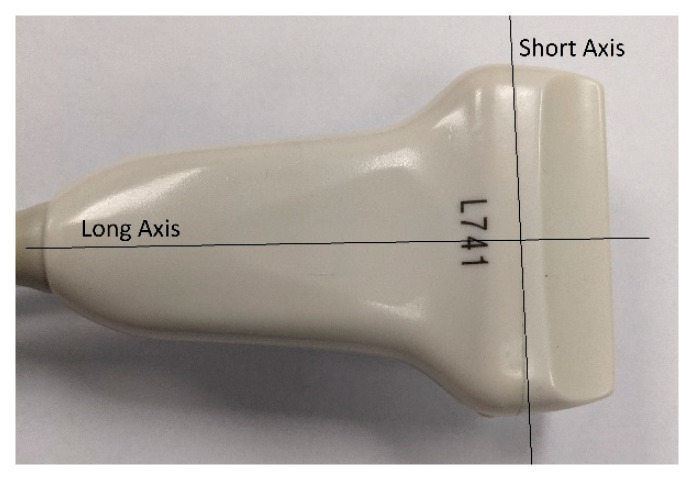
Ultrasound probe long and short axes.

The primary goal of this study was to evaluate the effect of the ultrasound probe position, probe and patient orientation, as shown in the Appendix A (Figure A1 and Figure A2 and Table A1), on the positions of small masses relative to the nipple and body and identify the key measurable features that determine the displacement of small breast masses during handheld breast ultrasound. Mass displacement represents the linear distance between a mass center point’s coordinate relative to the nipple and body orientation in two images obtained under different conditions of the body and probe position and rotation in a 3D coordinate system centered at the nipple. 

The secondary goals of this study include the evaluation of consecutive ultrasound operator ability to reproduce the position between exams using on-screen guidance and to quantify the time required to annotate and reidentify a breast mass when using a computer-assisted scanning device versus using the traditional manual method.

To the best of our knowledge, this is the first study to quantify the effects of changes in the body and handheld ultrasound probe positions on the displacement of small masses from their exact 3D location and to measure the difference in breast mass position annotations caused by operator variability.

## 2. Materials and Methods

### 2.1. Patients

This prospective study was approved by the Institutional Review Board (IRB) at Evanston Hospital (Evanston, IL, USA) and the IRB at Dacia Clinic (Oak Park, IL, USA). Written informed consent was obtained from all the participants. The patients were recruited from October 2018 to September 2020. The patients were eligible to enroll in this study if there was a documented presence of one or more breast masses that were palpable or non-palpable, benign or probably benign, less than 2 cm in maximum diameter and at least 4 cm away from the nipple. The 4 cm minimum distance to the nipple requirement was chosen to allow for ultrasound probe rotation without interfering with the nipple. Women with implantable electronic devices, known breast cancer, suspicious lesions, breast implants, and those who were pregnant or lactating were excluded from this study. Women with suspicious masses were not included to prevent interference with their clinical management, as the differences in morphological descriptors were not expected to affect the displacement of small masses in the breast. To evaluate the interoperator reproducibility, the effects of the ultrasound probe position, probe orientation and patient orientation changes on the displacement of small masses between exams were studied. Each participant underwent an ultrasound recording session with each of the two operators on the same day: two breast ultrasound recordings were acquired from each participant. 

A total of 28 subjects were included in this study (mean age ± SD = 49.21 ± 11.18 years, range = 26–70 years, all female) and 34 breast masses were identified and annotated (size range = 0.3–1.8 cm, average size = 0.92 cm, median size = 0.95 cm, 21/34 masses measured 1 cm or less). The demographics and clinical characteristics are summarized in Table 1.

### 2.2. Operators and Equipment

**Figure 1:**
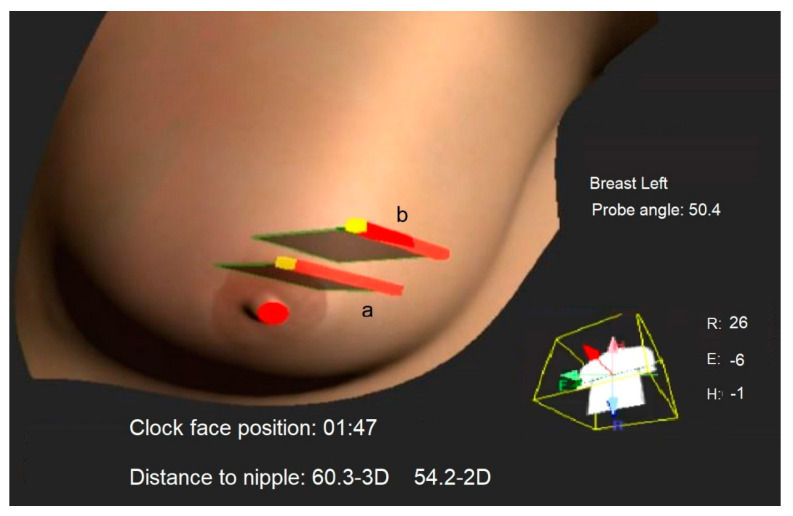
The 3D ultrasound frame position and orientation from a previous exam, labeled as ‘a’, is displayed as a gray rectangle. This is shown concurrently with the real-time ultrasound frame during the current exam, labeled as ‘b’. This approach assists consecutive operators in reproducing the 3D mass location by aligning frame ‘b’ with frame ‘a’. The probe head is represented by a red line, the orientation marker by a yellow dot and the nipple point by a red dot. Additionally, metrics about the current probe position relative to the nipple and body, as well as the body’s rotation on the exam table, are displayed in real-time for operator guidance.

An automated breast ultrasound mapping device, the DEVICE (BVN (Breast Volume Navigator) Model-G1000 system (MetriTrack, Inc., Hillside, IL, USA)), was FDA-cleared for clinical use, tested and validated in laboratory studies. The system provides automated, accurate and precise 3D position mapping for ultrasound images in laboratory testing, with less than 2 mm of error [10]. The DEVICE is an add-on to existing ultrasound machines and provides a separate touch screen for image annotation, 3D mapping and navigation on 3D breast maps. Magnetic sensors, which are attached to the patient’s skin and the ultrasound probe, are used to continuously monitor the nipple position, body rotation on the exam table, probe position and orientation coordinates (Figure 1).

The DEVICE was attached to an ultrasound machine equipped with a linear transducer (Logiq 9/ML6-15 and Logiq-E/12L-RS, GE Healthcare, Boston, MA, USA). Breast ultrasound examinations were performed by qualified medical imaging personnel: board-certified radiologists or sonographers certified by the American Registry for Diagnostic Medical Sonography, with a minimum of 3 years of experience in breast ultrasound. Five radiologists and three sonographers participated in this study and each of them was trained to use the DEVICE with breast phantoms for 3 to 4 h prior to scanning the patients. 

### 2.3. Scanning Protocol and Measurements

To evaluate the reproducibility of the mass location, the ultrasound probe position and orientation and patient orientation were systematically varied and precisely recorded by the DEVICE according to the protocol described below. The displacement of small masses during scanning, relative to the nipple and body axes, was studied. Each participant underwent two ultrasound recording sessions with two operators. Each operator manually annotated the position of the identified breast masses by marking the distance to the nipple (DN), clock face position (CFP) and probe orientation, according to the ACR recommendations [11,12], with the automated mapping device turned off.

**Figure 2:**
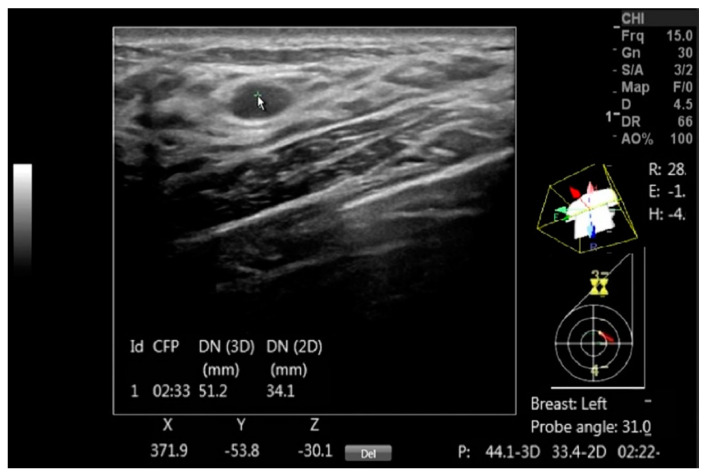
A DICOM image with a benign mass is annotated using DEVICE to document the mass, with the arrow pointing to the mass. The on-screen information includes the clock face position (CFP), the 3D distance to the nipple (DN (3D)), its projection in the coronal plane (DN (2D)) and the Cartesian coordinates (X, Y, and Z) for each mass center point. Additionally, the display shows the probe head orientation over the diagram, the probe axis tilt in degrees and the body rotation relative to the exam table, which are marked as R, E and H and given in degrees.

The DEVICE setup for automated breast mapping took less than a minute. By pointing to the center of a mass, automated positional data, including the mass center coordinates, probe position and rotation, and body rotation are instantly generated (Figure 2). 

The recorded metrics for each image are listed in Appendix A (Table A2 and Table A3). The high-resolution positional data recorded in the ultrasound images in increments of 1 mm and 1 degree was needed to investigate the different predictors of mass movement in the breast and to provide on-screen guidance for the second operator. However, such precise positional data are not intended to replace the ACR annotations format in clinical exams, where the DEVICE can round the values and annotate images using the standard ACR format showing the DN in 1 cm increments and the CFP in 0.5 or 1 h increments.

Multiple ultrasound images of the same breast mass were obtained by the first operator with the patient in a flat supine and semi-oblique position at an approximately 30-degree angle, with the ipsilateral arm raised overhead. Two representative orthogonal ultrasound images of the breast mass were obtained for each of the supine and semi-oblique patient positions. In addition, for each of the two probe head orientations, 2 additional ultrasound images of the mass were obtained by tilting the ultrasound probe long axis on each side by about 25 degrees relative to the corresponding initial image to change the direction of the deforming force caused by the probe pressure, for a total of 12 images per operator.

**Figure A3:**
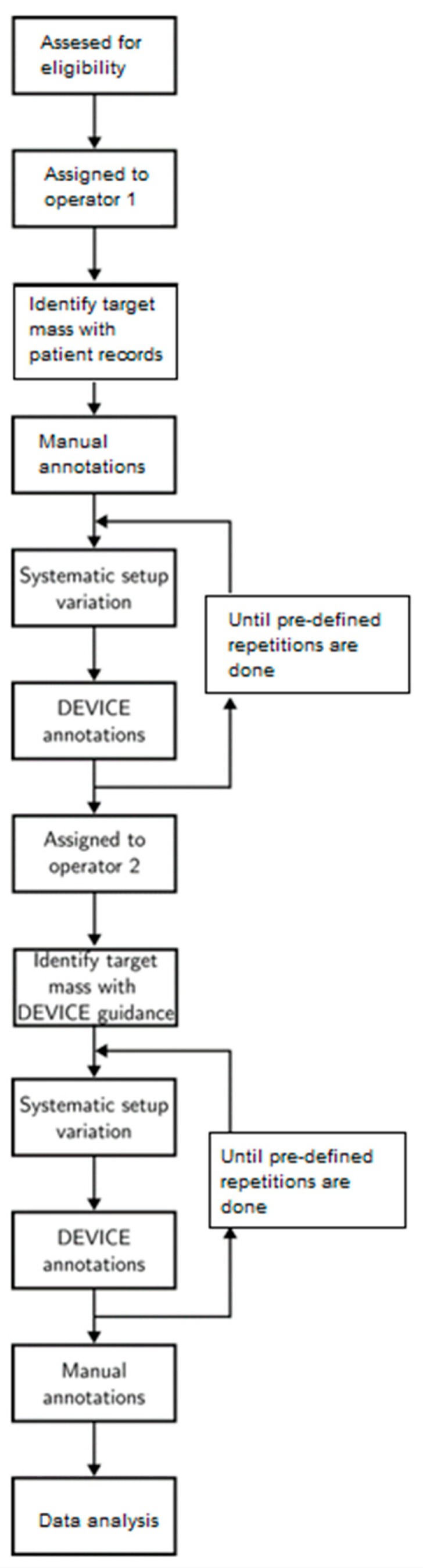
Study design.

In a new scanning session, the second operator reproduced the orthogonal images of each mass by aligning the real-time representation provided by the DEVICE of the probe scan plane with the previous exam baseline images of the first operator over the 3D breast map (Figure 1), with the patient in supine and semi-oblique positions. Subsequently, the ultrasound probe position and orientation were varied similarly to operator 1 without further assistance from the DEVICE, for a total of 12 images per patient. The scanning sessions were video recorded and time stamps were used to measure the time to annotate images and to find the masses. The study design is summarized in Figure A3 in Appendix A.

### 2.4. Data Acquisition and Analysis

The 3D distance or displacement between the mass center or probe head center points in two images, and their projections in the coronal plane, or the 2D displacement, were used to quantify the changes in mass and probe center location. The effect of the probe position and orientation and patient positioning changes relative to the exam table on the breast mass linear displacement were assessed using ultrasound images for a single mass from either a single operator (intraoperator) or from both operators (interoperator). The recording of a set of DEVICE measurements (see Appendix A) is fully automated and merely requires the operator to point on the device’s dedicated tactile screen. Operators were instructed to obtain 12 sets of DEVICE measurements under different probe and body position conditions for each mass.

Intraoperator pairs of DEVICE measurements

For each breast mass, all possible pairs of measurements of the mass in different images and by the same operator were collected, resulting in 2166 image pairs for analysis.

Interoperator pairs of DEVICE measurements

All possible pairs of measurements from the same mass but different operators were obtained, resulting in 3563 image pairs for analysis.

Data exclusion

Data were excluded from the analysis if the probe displaced the nipple sensor at the time that the image was taken or if the scanning protocol was not followed by the operator. To prevent mapping errors, data were also excluded if the absolute value of the chest rotation between the supine and semi-oblique positions was greater than 35 degrees, or if the absolute value of the patient long axis rotation with respect to the exam table in the horizontal plane exceeded 5 degrees during scanning. Consequently, 64 images were excluded out of the 857 acquired ultrasound images.

Logistic regressions

A list of 19 features (or parameters) were calculated to provide information about the patient positioning, probe location and orientation, and breast mass (see Table A2, Table A3 and Table A4 in Appendix A). The mass displacement was binarized using a 10 mm threshold to separate good (displacement < 10 mm) from poor reproducibility (displacement ≥ 10 mm). This threshold was selected, as it was found to be clinically significant for confident lesion localization [13]. The features were further divided into operator controllable or non-controllable, i.e., breast specific, groups (see Table A2 and Table A3). Controllable parameters could be directly modified by the operator and included parameters associated with the patient’s position or related to the ultrasound probe position and orientation. Non-controllable parameters were independent from the operator and included breast mass characteristics, such as its distance to the nipple or volume.

To identify the key parameters for successful exam reproducibility and to disentangle the importance of controllable and non-controllable parameters, three logistic regression models were implemented. 

The intraoperator model used only controllable parameters. Its purpose was to investigate the importance of each controllable feature on the mass displacements during scanning by a single operator. A total of 13 features were used as inputs. The interoperator model used the same controllable variables as in the intraoperator model but with pairs of images from different operators. Its purpose was to assess whether the controllable parameters could predict the reproducibility of the breast masses’ positions across operators and identify the most important parameters. The interoperator and anatomy model used the same features as in the interoperator model plus six additional features specific to the breast mass. This model aimed at evaluating whether the addition of non-controllable features substantially changed the predictability of the breast ultrasound exams’ reproducibility. The list of features used in each model can be found in Table A4 of Appendix A.

The models were trained using a 4-fold cross-validation approach that involved patient-wise shuffling to avoid data leakage, with normalized features and regularization with Elastic Net to prevent overfitting and improve performances with correlated features [14]. Feature selection was done using an extensive search that tried all feature combinations. The feature combination yielding the greatest average area under the curve (AUC) from the receiver operating characteristic (ROC) over the 4 folds was considered the best. The feature importance was determined by their Shapley values averaged over the folds [15]. The Shapley values provided the contribution of each selected feature to the prediction of the breast mass displacement such that important features were assigned large Shapley absolute values.

### 2.5. Comparison between Manual and DEVICE Annotations

Intraclass correlation coefficients (ICCs) [16] were calculated to quantify the interoperator reliability on the CFP and the DN corresponding to either manual or automated annotations of the probe head center, which were measured for each mass when the probe and body positions were matched. ICC values less than 0.5, between 0.5 and 0.75, between 0.75 and 0.9, and greater than 0.90 were indicative of poor, moderate, good and excellent reliability, respectively [16]. The time required by the second operator to find a small mass with the DEVICE on-screen guidance was recorded from the moment the probe touched the skin until the mass was first seen. The time required to record manual annotations with and without the DEVICE was compared. 

The displacement of the nipple point in each image relative to the first image was calculated during each session. The mass center position differences in the automated CFP and the DN measurements were calculated between the supine and rotated body positions for each scanning session to study the effect of body rotation. Finally, the influence of the mass laterality, i.e., medial vs. lateral quadrants, on the intra- and interoperator displacement was assessed using a linear model.

## 3. Results

### 3.1. Factors Predictive of Good Exam Reproducibility

**Figure 3:**
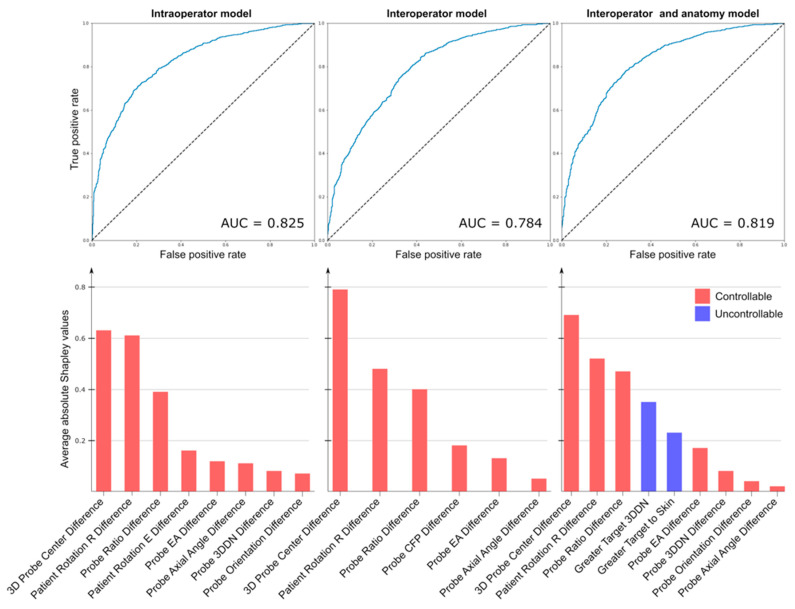
The performances of the three logistic regression models are illustrated with their ROC curves and the corresponding AUC values. For each model, the features were selected using an exhaustive search and were ranked based on their importance in the classification. The intraoperator model selected 8 features, while the interoperator model selected 6, and the interoperator and anatomy model selected 9 features. The three most important features were identical across all models.

The mass center displacement values from all paired images that represented the entire range of breast deformation states sampled in this study ranged between 4 and 47 mm for the interoperator measurements, where 2245 pairs (63%) showed displacements over 10 mm, and 3 to 45 mm for intraoperator measurements, where 1083 pairs (50%) showed displacements over 10 mm. The results of the three logistic regression models, along with the importances of the selected features, are shown in Figure 3. 

**Figure 4:**
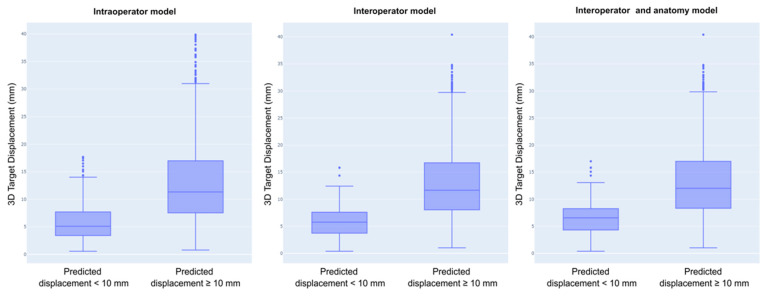
Each model displayed the actual displacement observed in the predicted classes. Across all models, the displacement of the samples that were classified as being well reproduced was significantly smaller compared with the displacement of other samples (*p* < 10^−10^).

All models discriminated small from large breast mass displacement with good and comparable performances, with the AUC being 0.825 for the intraoperator model, 0.784 for the interoperator model, and 0.819 for the interoperator and anatomy model. Although the feature selection step, which was performed using an exhaustive search, provided different sets of features across the models, the three most important features were identical in all models. The differences in 3D probe center, patient rotation R, and probe compression represented by the probe ratio difference were consistently the main drivers for the prediction of a large or small breast mass displacement in all models. The probe ratio was calculated by dividing the probe center distance to the nipple in the 3D space to its projection in the patient’s coronal plane, which depended on the amount of probe compression. These three main drivers accounted for 75% of the total Shapley values in the intraoperator model, 82% in the interoperator model, and 65% in the interoperator and anatomy model. The addition of breast mass non-controllable features improved the performance of the interoperator model, although rather modestly (interoperator model AUC = 0.784 and interoperator and anatomy model AUC = 0.819). Figure 4 illustrates the box plots of the actual displacement in the predicted classes. The displacements of the samples according to their classification are summarized in Table 2.

For all models, the displacement of the samples that were classified as being well reproduced was significantly smaller than that of the other samples (Mann–Whitney tests, *p* < 10^−10^).

The average change in the nipple point position relative to the body from the beginning to the end of each session was 81.0 ± 22.0 mm, with the largest changes observed when the patient’s position was altered from supine to semi-oblique. The automated CFP, DN and mass center differences between the two ultrasound operators when the patient’s body was supine or rotated are shown in Table 3. 

### 3.2. Comparison between Manual and DEVICE Annotations

**Figure 5:**
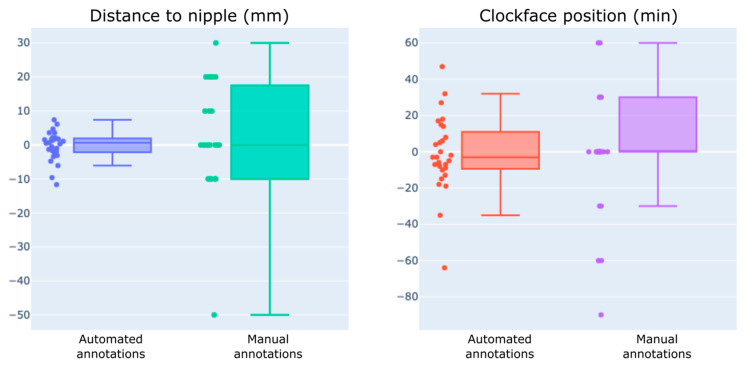
Differences in the distance to the nipple and clock face position between operators were assessed to compare the measurements obtained manually with those acquired with the DEVICE.

Using manual annotation, the interoperator ICC was 0.701 (95% confidence interval (CI95) 0.45–0.85) for DN and 0.988 (CI95 0.97–0.99) for CFP. Using the automated annotation, the interoperator ICC was 0.968 (CI95 0.93–0.99) for DN and 0.992 (CI95 0.99–1.00) for CFP. The interoperator differences between either the manual or automated annotations were 11.1 ± 11.6 mm and 3.1 ± 2.8 mm for the DN and 24.2 ± 28.2 min. and 14.9 ± 14.6 min. for the CFP, respectively (Figure 5). 

The linear displacement between the probe head centers was 8.2 ± 6.3 mm for the automated annotations and 19.9 ± 16.1 mm for the manual annotations. The mass center displacement between the operators using automated annotations was 6.6 ± 2.8 mm. 

The average time to identify a mass by the first operator and to reidentify the same mass with DEVICE guidance by the second operator was 57.0 ± 51.0 s and 11.0 ± 7.0 s, respectively. On average, the time to manually mark a breast mass was 40.0 ± 9.8 s and was 7.0 ± 3.0 s using automated annotations. The displacement of masses located in the lateral quadrants was not different from that of masses in the medial quadrant for both the intraoperator (*p* = 0.9) and interoperator data (*p* = 0.7). 

## 4. Discussion

Consistent recognition and characterization of lesions are critical in whole breast ultrasound applications and require the consistent reporting of lesion location, size and description of features [6]. Interobserver agreement for lesion classification and detection was significantly lower for dense breasts compared with non-dense breasts, with kappa κ = 0.55 and κ = 0.82, respectively [17]. Lesion size is another factor that affects lesion reproducibility in ultrasound, particularly for subcentimeter lesions. Interobserver agreement on several descriptors, including echogenicity, margin and posterior enhancement, is limited for subcentimeter lesions [6,8]. In one study, the BI-RADS category agreement was worse for lesions smaller than 0.7 cm (κ = 0.37) than for larger masses (κ = 0.67) [6], and another study showed the inconsistent characterization of cysts smaller than 8 mm in diameter [9]. Most lesions found during whole-breast ultrasound (89% [9] and 65% [6]) were less than 9 mm in size. In patients with multiple small masses, their identification across readers can be difficult and identifying the precise location of small masses between exams can help ultrasound users [18]. The landmark study ACRIN 6666 showed that 19.5% of patients required follow-up exams for probably benign masses; however, 38.7% of these masses were not present at the follow-up examinations [19], and approximately 25% of the probably benign masses were new and identified in subsequent ultrasound exams. Since most of the probably benign masses were complicated cysts, it is likely that some complicated cysts resolved, while others probably developed between the ultrasound exams.

The reproducibility of breast lesions between exams depends on multiple factors, including the gain settings and differences in probe compression [8]. The location reproducibility of lesions in the breast is important for overall exam reproducibility. The representation of lesion location using clock face position annotations in 30 min increments and the distance to the nipple in cm rather than the quadrant and the A, B, C classification for distance to the nipple can facilitate the follow-up of lesions [8]. However, the routinely used reporting of lesion position in the breast using 2D polar coordinates, the CFP and DN does not account for probe compression and is subject to errors due to visual estimation and breast deformation. In this study, three logistic regression models were trained to discriminate well-reproduced from poorly reproduced exams, as defined by a 10 mm cutoff on 3D breast mass displacements. For all models, the displacement of small breast masses was mostly driven by three controllable features: the 3D probe center position, patient body rotation and probe compression. By controlling these features using on-screen guidance, the interoperator and intraoperator models show that the AUCs for successful mass position reproducibility could reach similarly high values of 0.78 and 0.82, respectively. These study results confirm that matching the operator controllable features can largely eliminate the interoperator variability in reproducing the location of small masses between exams. When added to the interoperator logistic regression model, the mass-dependent variables modestly improved the AUC from 0.78 to 0.82, with the DN and distance to skin being the most important. There was no significant difference in the magnitude of the mass displacements between the lateral and medial breast quadrants, which indicates the ability to precisely localize small masses throughout the breast when the main controllable variables are monitored. The probe head orientation difference was not found to be a main driver for mass displacements, which indicates the ability to reproduce the breast deformation by only matching the probe force vector direction and magnitude, as well as the body position.

**Figure 6:**
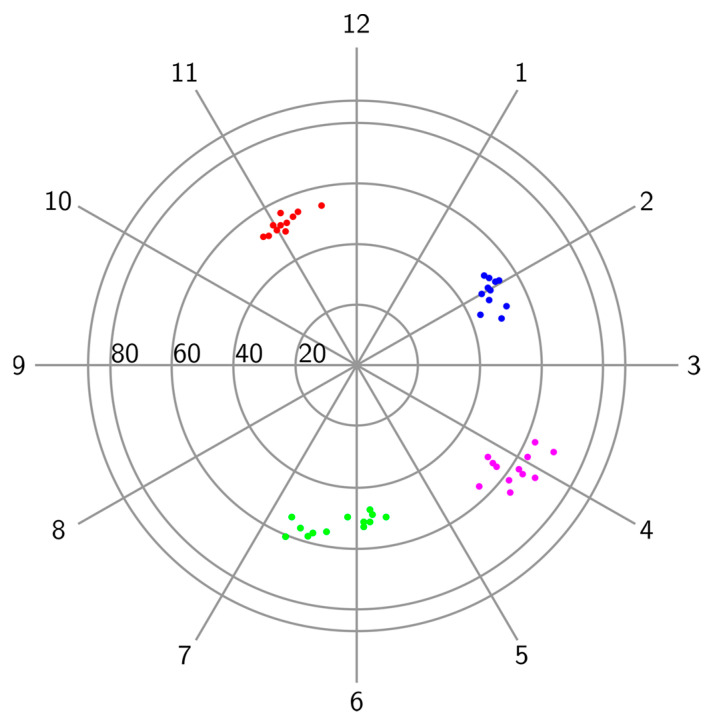
The distribution of mass displacements across four subjects, one in each quadrant, is illustrated using clock face positions (hours) and distances to the nipple (mm). The colored dots on the figure represent the positions of four masses across repeated recordings. The dots with the same color represent the positions of the same mass.

This study showed that breast deformation due to changes in the patient’s orientation on the exam table and probe position encountered during ultrasound scanning could cause mass displacements that spanned over several centimeters (Figure 4). The interoperator mass center displacements were larger when the patients’ body rotation was not matched between exams, with 14.9 ± 7.1 mm, than for the exams performed with the patient in the same position on the exam table, with 9.1 ± 4.7 mm, as seen in Table 3. This underlines the importance of matching the body rotation during repeat exams to obtain good mass location reproducibility. At the same time, the mass DN was stable regardless of the breast deformation caused by body rotation or probe position changes, with differences between operators of less than 5 mm on average, as seen in Table 3. Published data show that the DN is the most stable measurement during breast deformation, with a variation of 0.7 ± 0.2 cm between prone MRI and supine ultrasound [20]. More variability was seen with the CFP, which can explain the movement of small masses along an arc centered on the nipple, as seen in Figure 6, as previously reported [21].

The knowledge of this distribution pattern can be used with the on-screen navigation feature provided by the DEVICE to narrow the searching area during second-look ultrasound exams, thus increasing the confidence in identifying MRI detected lesions with ultrasound. 

There was an excellent interoperator reliability for the probe CFP and DN with the automated annotations (ICC 0.99 and 0.97, respectively). The manual interoperator reliability for the DN was moderate (ICC = 0.7), with an average difference of 11.1 ± 11.6 mm, which was possibly due to the effect of visual estimation. Excellent reliability was found for the manual CFP (ICC = 0.99). The obtained ICC values for manual annotations were in line with previous studies [6,8], where good reliability for the DN and CFP were obtained manually. However, despite the good interoperator reliability found for the manual annotations, the associated linear displacement was significantly larger than for the automated measurements of the same mass, with 19.9 ± 16.1 mm and 8.2 ± 6.3 mm, respectively. 

This study’s results show that the real-time mapping and display of prior and current images referenced to the nipple and body orientation over 3D maps of the breast can help ultrasound operators to precisely align images between exams and control the main features needed to reproduce the 3D locations of small masses to within less than 10 mm. In addition, the 3D probe guidance capability can help ultrasound operators to match the image depth relative to the nipple and, therefore, probe compression between exams, and at the same time, it helps to compare the surrounding breast anatomy. The results obtained using the 3D mapping of lesions and ultrasound frames described above suggest the potential for overall improved lesion reproducibility compared with the currently used position annotations and probe orientation representation in a polar 2D coordinate system. 

The precise mapping of small breast lesions is crucial for identifying them during biopsy and surgery [18,19], as well as for serial follow-ups to monitor the stability or response to treatment. Ultrasound can evaluate breast tumor size at diagnosis with the same accuracy as MRI, with 87.7%, for women treated with breast-conserving surgery [22] and it has been shown to be superior to MRI in assessing the size of residual tumors after neoadjuvant chemotherapy [23]. Since the pathological complete response to neoadjuvant chemotherapy cannot be reliably evaluated with current imaging modalities, surgical removal of the entire tumor area is often mandated [24]. Disease-free margins of resection are an important predictor of clinical outcomes [23]. The precise 3D mapping and display of tumors could provide tailored guidance for placing preoperative markers, thereby aiding in planning and performing breast surgery.

The automated position annotation values can be rounded to match the usual format used by ultrasound operators, as recommended by the ACR. Meanwhile, the high-resolution precise probe and body coordinates can also be stored. These features assist operators during subsequent exams performed without DEVICE guidance, while precise on-screen guidance is available for the follow-up of images obtained with the DEVICE.

Manual annotations require multiple repetitive steps that involve complex hand movements that can be time consuming and cause fatigue. Some new ultrasound machines have auto-protocol annotation capabilities; however, this approach continues to rely on the operator’s subjective input and can still be labor intensive. Automated position annotations were significantly faster to generate than manual annotations, with 7.0 ± 3.0 s vs. 40.0 ± 9.8 s, respectively, which is in line with the published literature [25], and foster reduced scanning times, especially when multiple lesions are present. The identification of a previously mapped mass by the second operator with on-screen guidance was faster than without using the DEVICE, which can save additional time during follow-up exams. It took 11.0 ± 7.0 s compared with the 57.0 ± 51.0 s spent by the first operator to find a mass using a previous exam report and images. 

The standardization of scanning and image acquisition protocols is supported by the generation of automated annotations and the precise 3D mapping of images with computer-assisted scanning, which, in turn, is needed for the successful implementation of artificial intelligence (AI) tools to further decrease the interobserver variability and decrease false positive rates [26]. Ultrasound technologists, especially those whose practice is not solely devoted to breast imaging, could benefit from standardized mapping, as they can more easily generate images with complete and precise annotations for an improved accuracy of interpretation, as well as reproducibility of lesion localization at follow-up, thus benefiting radiologists and technologists alike. This technology has the potential to be instrumental in reducing training time for performing ultrasound exams with improved standardization, thus increasing productivity, quality and safety, which is particularly advantageous given the progressively worsening shortage of breast ultrasound technologists and radiologists.

There were several limitations of this study. The number of subjects was relatively small and additional data are needed to further study the effects of mass-specific variables, like the size, shape and position, in the breast on the displacement of breast masses. Most subjects had a dense parenchymal pattern and the displacement of small masses in fatty breasts may be different than in dense breasts. There is also potential selection bias due to the presence of already known breast masses and the exclusion of subjects with suspicious masses or known cancer to prevent interference with their clinical management. The study scanning protocol was different from the routine exam protocols as needed to generate the study data. 

However, despite the above limitations, this study confirmed the highly positive contribution of real-time computer-assisted 3D mapping to HHUS by increasing the lesion location reproducibility and improving the time efficiency over the traditional handheld exams. The results of this study support the feasibility of a larger study to further evaluate the advantages of automated mapping for the overall reproducibility of breast lesions in the clinical workflow and improvement in performance, especially for non-dedicated breast sonographers.

## Figures and Tables

**Table 1:** Demographics and clinical summary.

Subject Age, Mean ± SD (Range)		49.21 ± 11.18 Years (26–70)
Number of masses per subject, total *N* (%)		
	1	25 (87%)
	2	3 (10%)
	3	1 (3%)
Breast mass sizes, mean (range)		0.92 cm (0.3–1.8)
Mass type, total *N* (%)		34 (100%)
	Simple cyst	14 (42%)
	Complicated cyst	11 (32%)
	Solid	9 (26%)
Palpable, *N* (%)		
	Yes	5 (15%)
	No	29 (85%)
Quadrant location, *N* (%)		
	UOQ	11 (32%)
	UIQ	15 (44%)
	LIQ	4 (12%)
	LOQ	4 (12%)

Upper outer quadrant (UOQ); Upper inner quadrant (UIQ); Lower inner quadrant (LIQ); Lower outer quadrant (LOQ).

**Table 2:** Median and interquartile range (IQR) of the displacement of the samples that were classified as being well reproduced (displacement < 10 mm) or not.

Model	Predicted Displacement < 10 mm (Median [IQR])	Predicted Displacement ≥ 10 mm (Median [IQR])
Intraoperator	5.09 mm [3.42–7.70]	11.32 mm [7.54–16.98]
Interoperator	5.76 mm [3.73–7.59]	11.66 mm [8.04–16.72]
Interoperator and anatomy	6.54 mm [4.31–8.25]	12.00 mm [8.32–17.00]

**Table 3:** Average interoperator difference of CFP, 3D DN and 3D mass center displacement with automated annotations.

Body Position for Both Operators	CFP (min)	DN (mm)	3D Displacement (mm)
Both supine	9.0 ± 11.4	4.0 ± 3.6	9.1 ± 4.7
Both semi-oblique	12.3 ± 9.5	3.6 ± 3.2	11.8 ± 5.4
Supine and semi-oblique	14.3 ± 13.6	4.4 ± 3.3	14.9 ± 7.1

## Data Availability

The data presented in this study are available on request from the corresponding author due to privacy restrictions.

## Appendix A

**Table A1:** List of the landmarks used by the DEVICE to extract the relevant anatomic information and provide feedback to the operator.

Landmarks	Description
xy plane	Patient’s coronal plane
O	Nipple sensor
T	3D target (breast mass) center coordinates
P	3D probe head center point coordinates
$T^{\prime }$	Orthogonal projection of T in the xy plane
$P^{\prime }$	Orthogonal projection of P in the xy plane
N	12 o’clock clockwise face position

**Table A2:** List of operators related DEVICE measurements.

DEVICE Metrics	Description	Controllable
R	Patient’s transverse axis (*x*) rotation with respect to the transverse axis of exam table in the transverse plane	Yes
H	Patient’s long axis (*y*) rotation with respect to the table’s long axis in the horizontal plane	Yes
E	Patient’s long axis (*y*) rotation with respect to the table’s long axis in the sagittal plane	Yes
β	Probe axial angle, angle between the probe’s long axis and z-axis	Yes
EA	Probe Euler angles (roll, pitch, yaw)	Yes
α	Probe orientation angle, i.e., angle between the probe’s short axis and the y-axis projected in the *xy* plane	Yes
P3DDN	Probe head center point 3D distance to nipple $\left|OP\right|$	Yes
P2DNN	Probe head center point distance to nipple projected in xy plane (2D) $\left|{OP}^{\prime }\right|$	Yes
P_ratio	Ratio between probe 3DNN and probe 2DDN	Yes
P_height	Distance from probe center point to nipple in z axis $\left|{PP}^{\prime }\right|$	Yes
P_CFP	Probe clockface position, i.e., angle $\hat{P^{\prime }ON}$	Yes

**Table A3:** List of breast-mass-related DEVICE measurements. These measurements can be accessed only after a breast mass has been identified and marked.

DEVICE Metrics	Description	Controllable
T3DDN	Target center point 3D distance to nipple $\left|OT\right|$	No
T2DDN	Target center point distance to nipple projected in xy plane (2D) $\left|OT^{\prime }\right|$	No
T_ratio	Ratio between target 3DDN and target 2DDN	No
T_CFP	Target clockface position, i.e., angle $\hat{T^{\prime }ON}$	No
T_height	Distance from target center point to nipple in z-axis $\left|TT^{\prime }\right|$	No
T_skin	Distance between target center point and skin $\left|PT\right|$	No
T_chest	Distance between target center point and chest surface $\left|TC\right|$	No
T_skin_chest_ratio	Ratio between target to skin and target to chest	No
T_diameter	Target’s longest diameter as measured on the ultrasound image	No
T_volume	Target’s volume measured using an ellipsoid fitting on the 3 main axes	No

**Table A4:** List of features used in the logistic regression models. These features were obtained using two sets of DEVICE measurements from paired mass images when the same mass was measured in different conditions. For the intraoperator analysis, the pair of measurements were obtained from the same operator, whereas for the interoperator analysis, they were obtained by 2 different operators. The target displacement threshold of 10 mm was used as the binary label of logistic regression models to indicate whether a displacement was large, and all other features were used as predictors.

Features	Description	Controllable
Patient rotation R difference	$\left|R_{1}-R_{2}\right|$	Yes
Patient rotation E difference	$\left|E_{1}-E_{2}\right|$	Yes
Patient rotation H difference	$\left|H_{1}-H_{2}\right|$	Yes
Probe axial angle difference	$\left|\beta_{1}-\beta_{2}\right|$	Yes
Probe EA difference	$\hat{{\mathrm{EA}}_{1}{\mathrm{EA}}_{2}}$ †	Yes
Probe orientation difference	$\left|\alpha_{1}-\alpha_{2}\right|$	Yes
2D probe center difference	$\|\vec{{P^{\prime }}_{1}{P^{\prime }}_{2}}\|$	Yes
3D probe center difference	$\|\vec{P_{1}P_{2}}\|$	Yes
Probe height difference	$\left|{P\_\mathrm{height}}_{1}-{P\_\mathrm{height}}_{2}\right|$	Yes
Probe CFP difference	$\left|{P\_\mathrm{CFP}}_{1}-{P\_\mathrm{CFP}}_{2}\right|$	Yes
Probe ratio difference	$\left|{P\_\mathrm{ratio}}_{1}-{P\_\mathrm{ratio}}_{2}\right|$	Yes
Probe 2DDN difference	$\left|{P2\mathrm{DDN}}_{1}-{P2\mathrm{DDN}}_{2}\right|$	Yes
Probe 3DDN difference	$\left|{P3\mathrm{DDN}}_{1}-{P3\mathrm{DDN}}_{2}\right|$	Yes
Greater target to skin	$max\left(T\_skin_{1},\;T\_skin_{2}\right)$	No
Greater target to chest	$max\left(T\_chest_{1,}T\_chest_{2}\right)$	No
Greater target 3DDN	$\max\left({T3\mathrm{DDN}}_{1},{T3\mathrm{DDN}}_{2}\right)$	No
Greater target 2DDN	$max\left(T2DDN_{1},T2DDN_{2}\right)$	No
Greater target CFP	$\max\left({T\_\mathrm{CFP}}_{1},{T\_\mathrm{CFP}}_{2}\right)$	No
Greater lesion volume	$\max\left({T\_\mathrm{volume}}_{1},{T\_\mathrm{volume}}_{2}\right)$	No
Target displacement	$\|\vec{T_{1}T_{2}}\|$	

† The angle between two sets of Euler angles was calculated by determining the angular distance between corresponding quaternions.
